# The Great Physician; the Connexion of Diseases and Remedies with the Truths of Revelation

**Published:** 1843-01

**Authors:** 


Art. VIII.—*The Great Physician; the Connexion of Diseases and Remedies with the Truths of Revelation.* By John Gardner, Surgeon.—*London,* 1843. 8vo, pp. 359.

With the exception of the last chapter, which contains "a brief history of epidemic diseases, or pestilences," the contents of this volume are exclusively of a theological character, and come not, therefore, within our jurisdiction. When the second part is published, which will principally consist of medical matters, we may return to the work. The author is evidently a very sincere and pious person, but we suspect that he is too enthusiastic a religionist to deal philosophically with such a subject.

